# Genome Editing for β-Hemoglobinopathies: Advances and Challenges

**DOI:** 10.3390/jcm10030482

**Published:** 2021-01-28

**Authors:** Giacomo Frati, Annarita Miccio

**Affiliations:** Laboratory of Chromatin and Gene Regulation during Development, Imagine Institute, Université de Paris, INSERM UMR 1163, F-75015 Paris, France

**Keywords:** genome editing, β-hemoglobinopathies, gene therapy

## Abstract

β-hemoglobinopathies are the most common genetic disorders worldwide and are caused by mutations affecting the production or the structure of adult hemoglobin. Patients affected by these diseases suffer from anemia, impaired oxygen delivery to tissues, and multi-organ damage. In the absence of a compatible donor for allogeneic bone marrow transplantation, the lifelong therapeutic options are symptomatic care, red blood cell transfusions and pharmacological treatments. The last decades of research established lentiviral-mediated gene therapy as an efficacious therapeutic strategy. However, this approach is highly expensive and associated with a variable outcome depending on the effectiveness of the viral vector and the quality of the cell product. In the last years, genome editing emerged as a valuable tool for the development of curative strategies for β-hemoglobinopathies. Moreover, due to the wide range of its applications, genome editing has been extensively used to study regulatory mechanisms underlying globin gene regulation allowing the identification of novel genetic and pharmacological targets. In this work, we review the current advances and challenges of genome editing approaches to β-hemoglobinopathies. Special focus has been directed towards strategies aimed at correcting the defective β-globin gene or at inducing fetal hemoglobin (HbF), which are in an advanced state of clinical development.

## 1. Introduction

Hemoglobinopathies are the most frequent monogenic diseases worldwide, with approximately 400,000 affected births each year [1]. The most common hemoglobinopathies are β-thalassemia and sickle cell disease (SCD). Patient affected by β-thalassemia show low or absent production of adult β-globin chains; this leads to α-/β-globin chain imbalance, apoptosis of erythroid cells, hemolysis and iron overload. In SCD, a single point mutation causes the β7^Glu→Val^ substitution that leads to the production of a mutant β-globin chain (β^S^). Once incorporated in the hemoglobin tetramer (HbS), the valine in position 7 confers to the Hb the propensity to polymerize. HbS polymerization causes red blood cell (RBC) sickling, hemolysis, iron overload, vaso-occlusive crises and multi-organ damage.

Palliative treatment consisting of lifetime blood transfusion combined with iron chelation, although costly, is commonly used to suppress anemia and reduce iron toxicity in β-thalassemia patients. SCD patients with a severe clinical phenotype are usually treated with RBC exchange transfusion and iron chelation. In developed countries, this standard treatment for β-hemoglobinopathies improves patients’ quality of life. However, these diseases can still result in fatal outcomes. In African countries, where SCD is endemic, the majority of children born with SCD do not reach adulthood [1]. Allogeneic hematopoietic stem cell transplantation (HSCT) is a curative option, but its success depends on the availability of a compatible human leukocyte antigen (HLA)-matched donor. Studies in large cohorts of β-thalassemia and SCD patients receiving identical sibling transplants showed 83% and 91% of disease-free survival, respectively, with better clinical outcomes in younger patients [2,3]. However, the chances of finding an HLA-matched sibling or related donor are limited [4]. Recently, it has been shown that unrelated HLA-matched transplantation is possible for β-thalassemia patients lacking of an HLA-matched sibling donor, when stringent criteria of HLA class I and II compatibility between the donor and the recipient are met. Despite the limited number of reported cases, results in β-thalassemia patients are encouraging, thus this approach represents a promising therapeutic option [5,6].

Gene therapy nowadays represents an important curative option for β-hemoglobinopathies. The transplantation of genetically modified, autologous HSCs avoids the immunological risks such as graft rejection and graft-versus-host disease associated with allogeneic HSCT and use of immunosuppressive drugs to prevent these complications. In the last decades, several research groups engineered lentiviral vectors (LV) to successfully transfer a functional β-globin chain in HSC-derived erythroid cells [7,8,9,10]. These paramount studies paved the way for several gene therapy clinical trials including more than 100 patients treated with LV-transduced bone marrow (BM)- or mobilized peripheral blood (mPB)-derived HSCs [11,12,13,14,15,16,17,18,19,20,21,22]. The majority of these patients showed a reduced transfusion requirement after gene therapy and this treatment can be considered reasonably safe. However, some patients, especially those with a more severe clinical phenotype, are still transfusion-dependent, because the optimal therapeutic Hb levels have not been achieved and/or because of the low gene transfer in HSCs. Current progresses and challenges of LV-mediated gene therapy approaches for β-hemoglobinopathies are reviewed elsewhere [23]. Briefly, the major limitations are: (i) the lack of an optimal protocol to reproducibly obtain a successful gene transfer in HSCs; (ii) the inability of LVs to sustain high β-globin expression at low vector copy numbers; (iii) the poor engraftment of transduced HSCs into the patient BM environment, which is still poorly studied and negatively affected by disease progression; (iv) the potential genotoxic risk associated with the semi-random integration of LVs [24].

In the last decade, the numerous discoveries in the field of genome editing have led to the development of novel and powerful strategies for the treatment of β-hemoglobinopathies. In particular, the efficiency of genome editing was greatly enhanced by the development of designer nucleases to generate DNA double strand breaks (DSBs) in a specific region of interest. Different nuclease-based editing strategies aimed either to correct the disease-causing mutations or to induce therapeutic levels of fetal Hb (HbF) have been proposed. The success of these strategies depends on the efficacy of the nucleases to target the DNA, the activity of the approach-specific cellular machinery repairing the DSB in HSCs and the capability of edited cells to stably engraft and provide high levels of therapeutic Hb. However, nucleases can trigger DSB-induced cytotoxicity and the formation of chromosomal rearrangements. The recent introduction of nuclease-free technologies has provided suitable solutions to overcome these issues and is likely shortening the way for the clinical translation of editing strategies for the treatment of β-hemoglobinopathies. The goal of this review is to give a comprehensive overview of the current advances and challenges of genome editing strategies for the treatment of β-hemoglobinopathies.

## 2. Globin Gene Regulation

The genes encoding for the human α- and β-like globin chains are located on chromosomes 16 and 11, respectively, and are arranged in the order of their expression during development. The α-globin cluster consists of three genes (ζ *[HBZ*], α2 [*HBA1*], and α1 *[HBA2*]), and the human β-globin cluster contains five genes (ε [*HBE1*], Gγ [*HBG2*], Aγ [*HBG1*], δ [*HBD*], and β [*HBB*]). Fetal Hb (HbF) is composed of two α- and two γ-globin chains. Fetal-to-adult Hb switching occurs soon after birth, and adult Hb (HbA) is composed of two α- and two β-globin chains. Several *cis*-regulatory elements are essential for the regulation of globin gene expression, including promoters and locus control regions (LCRs) containing DNase-hypersensitive sites (HSs). The α-globin LCR contains four enhancers, referred to as multispecies conserved sequences (R1 to R4). The β-globin LCR contains five HSs (HS1 to HS5); HS2, HS3, and HS4 are the main *cis*-regulatory regions involved in the stimulation of β-like globin gene transcription [25]. The β-globin LCR regulates the expression of the β-like genes via looping interactions with the β-like globin promoters [26].

## 3. Basics of Genome Editing Technologies

Nuclease-mediated genome editing is based on the generation of a DSB at the target site and its repair (with or without the incorporation of a donor sequence) via different cellular mechanisms. The success of genome editing strategies depends on the use of effective, precise and safe engineered nucleases. Homing nucleases, also known as meganucleases, pioneered the field; however, their complexity limited their development for therapeutic purposes. Zinc-finger nucleases (ZFN) are composed of three to six zinc-fingers that bind DNA sequences and a cleavage domain that needs to dimerize to generate DSBs. Despite their complexity, ZFNs were the first nucleases used in clinical trials [27]. Similarly, transcription activator-like effector nucleases (TALENs) harbor DNA binding and cleavage domains, and require dimerization to produce DSBs, but they are considerably easier to design, thus explaining their widespread use.

The introduction of clustered regularly interspaced short palindromic repeats (CRISPR)/Cas9 technology in 2012 has revolutionized the genome editing field. The major difference between this system and the previously developed nucleases is that the Cas9 nuclease is targeted to the DNA by an RNA molecule (guide RNA, gRNA) via Watson-Crick base pairing instead of a protein-DNA interaction. Cas9 activity requires the presence of protospacer adjacent motifs (PAMs), short sequences located downstream of the target sequence (protospacer) complementary to the gRNA. PAMs are specific for each Cas9 derived from the different bacterial species (e.g., NGG for the most widely used *Streptococcus Pyogenes* Cas9). Several cellular mechanisms exist to repair DSBs. The two broken ends can be directly joined together by non-homologous end joining (NHEJ) usually generating insertion and deletion (Indel) events that can lead to the inactivation of genes or *cis*-regulatory regions. Alternatively, a DNA donor template can be incorporated in the DSB via homology directed repair (HDR). Finally, the microhomology mediated end joining (MMEJ) or alternative non-homologous end joining (Alt-NHEJ) is an error-prone pathway using microhomology sequences to align the broken ends before joining, resulting in deletions flanking the DSB [28]. Interestingly, in the presence of a donor template, MMEJ can easily allow gene knock-in [29]. The choice of the DNA repair mechanism is influenced by the cell type and the phase of cell cycle. In particular, HDR is active almost exclusively during the S/G2 phase of the cell cycle, MMEJ during the M and early S phases and NHEJ is active throughout the cell cycle [30,31].

The CRISPR/Cas9 system is nowadays widely used because of its easy design and high efficiency. However, the use of CRISPR/Cas9 raises safety concerns that may impair its therapeutic application. If since the beginning possible off-target activity has been taken into consideration as a major limitation, more recent studies pointed out that an adaptive immune response against Cas9 protein can occur and a relevant fraction of individuals has anti-Cas9 pre-existing antibodies [32,33]. Furthermore, CRISPR/Cas9-induced DSBs can lead to chromosomal rearrangements, such as deletions, inversions and translocations [34,35,36,37]. Finally, several studies show that p53 pathway activation reduces CRISPR/Cas9 editing efficiency in human stem cells and impairs hematopoietic stem/progenitor cell (HSPC) proliferation and engraftment [38,39]. As these cells are the target population of gene therapy protocols, this may represent a serious barrier to overcome. Some of these issues can be minimized by using DSB-free base and prime editing systems.

Base editing is a newly developed tool able to edit DNA sequences in a specific locus inducing little or no DSBs [40]. Different base editors (BEs) have been generated allowing base conversions in a variety of target regions. The cytosine BEs (CBEs) allow the conversion of a C:G to a T:A bp, while adenine BEs (ABEs) convert an A:T into a G:C bp. BEs are composed of a catalytically dead Cas9 (dCas9) or a nickase Cas9 (nCas9) fused to a deaminase and guided by a gRNA to the locus of interest. The d/nCas9 recognizes the specific PAM sequence and the DNA unwinds thanks to the complementarity between the gRNA and the protospacer. Then, the opposite DNA strand is accessible to the deaminase, which converts the bases located in a specific activity or editing window.

Furthermore, a novel genome editing technology, known as prime editing (PE), has been recently developed. The PE system relies on a prime editing gRNA (pegRNA) that drives a nCas9 fused with a reverse transcriptase to a specific locus and includes a template carrying the desired edits that is reverse transcribed and inserted in the target site. Notably, PE is less constrained by the availability of PAMs compared to BE, which can be >30 bp far from the target site. PE is able to generate not only all 12 types of base conversions, but also insertions of up to 44 bp and deletions of up to 80 bp, and even combination of substitutions, insertions and deletions [41].

## 4. Nuclease-Mediated Strategies for β-Globin Gene Repair

### 4.1. HDR-Based Gene Correction Strategies

HDR is a high-fidelity DNA repair pathway based on the use of a DNA donor template harboring sequences homologous to the target region. An exogenous donor sequence can be used as a template for correcting disease-causing mutations. The choice of the optimal exogenous template is crucial for the establishment of an efficient HDR-based gene correction protocol. The desired donor sequence can be delivered in HSPCs by transfection as single-stranded oligodeoxynucleotide (ssODN) or by a viral vector such as integrase-defective LVs (IDLVs) or recombinant adeno-associated viral vectors serotype 6 (rAAV6). rAAV6 has been considered for a long time as the most efficient delivery system for HDR-mediated gene correction in HSPCs [39,42,43]. However, recent comparative studies showed that, although more efficient in vitro, rAAV6 treatment caused a reduced HSC engraftment in immunodeficient mice compared to ssODNs [44,45].

**Figure 1:**
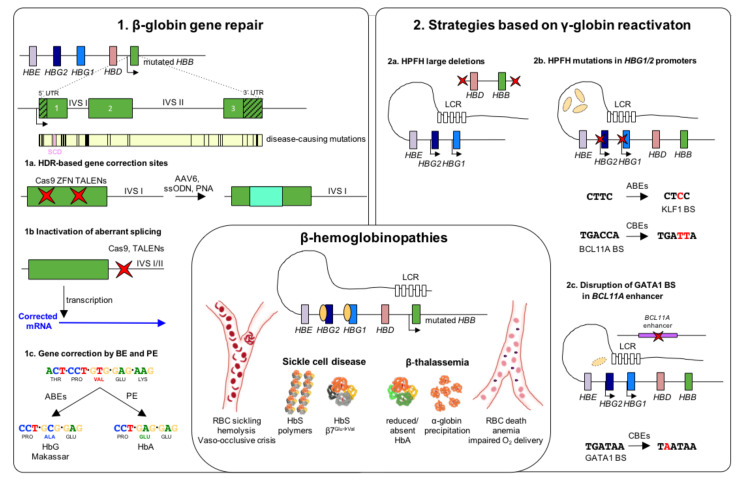
Genome editing strategies for β-hemoglobinopathies. β-hemoglobinopathy-associated variants in the β-globin locus cause the insufficient (β-thalassemia) or abnormal (SCD) expression of β-globin. Reduced β-globin expression leads to globin imbalance and α-globin precipitation causing RBC death, anemia and impaired oxygen delivery. In SCD, a single amino acid substitution (β7^Glu→Val^) confers to the hemoglobin (HbS) a higher propensity to polymerize under hypoxic conditions, leading to RBC sickling, hemolysis and vaso-occlusive crises. (**1**). Strategies based on β-globin gene repair. Schematic representation of the β-globin gene including 5′ and 3′ untranslated region (UTR) and a map of the most common β-hemoglobinopathy-causing mutations. (**1a**) Several genome editing approaches have been tested for repairing β-globin mutations through the homology direct repair (HDR) pathway. (**1b**) Non-homologous end-joining (NHEJ) has been used to target aberrant splicing sites in introns I and II, resulting in the restoration of functional β-globin transcripts. (**1c**) Newly-established base (BE) and prime editing (PE) approaches proved their efficacy in correct the SCD-causing mutation. In particular, adenine base editors (ABEs) are suitable to convert the GTG codon into a GCG triplet, leading to the production of a non-pathogenic Hb variant (HbG Makassar). Thanks to a pegRNA-guided reverse transcriptase activity, PE can revert the SCD mutation, restoring the production of WT hemoglobin (HbA). (**2**). Strategies based on γ-globin reactivation. (**2a**) Nuclease-mediated strategies aimed at reproducing natural occurring large deletions that reactivate γ-globin expression. These deletions could either eliminate HbF inhibitory sequences or juxtapose the γ-globin promoters to remote enhancer regions. (**2b**) Targeting the *HBG1/2* promoters with nucleases results in the disruption of binding sites (BS) for γ-globin transcriptional repressors, leading to a potent HbF expression. ABEs and cytosine base editors (CBEs) can be used to reproduce HPFH mutations that either create BSs for transcriptional activators (e.g., KLF1) or disrupt repressor BSs (e.g., BCL11A). (**2c**) Disruption of the GATA1 motif in the erythroid-specific intronic enhancer of *BCL11A* (achieved either using nucleases or CBEs) determines BCL11A knock-down and, consequently, reactivation of γ-globin expression. Red crosses indicate edits. LCR: locus control region; RBC: red blood cells; HbS, sickle hemoglobin; HbA, adult hemoglobin; IVS: intervening sequence; ZFN: zinc finger nuclease; TALENs: transcription activator-like effector nucleases; AAV6: adeno-associated viral vector serotype 6; ssODN: single-stranded donor oligonucleotides; PNA: peptide nucleic acid; HPFH: hereditary persistence of fetal hemoglobin.

Due to its monogenic nature and high prevalence, SCD is an ideal target for HDR-based gene correction. In the last decade, several strategies have been applied to revert the SCD-causing mutation. ZFNs, TALENs, and CRISPR/Cas9 have been proven successful in inducing HDR-mediated gene correction in induced pluripotent stem cells (iPSCs) [46,47] and SCD patient-derived HSPCs [43,45,48,49] (Figure 1). In HSPCs in vitro, the efficiency of correction ranged from 7% to 50%, depending on different editing tools and donor delivery systems. These frequencies were sufficient to produce clinically-relevant amount of HbA (up to 50% of total Hb) and to ameliorate the SCD cell phenotype in vitro [50]. However, xenotransplantation experiments showed that the gene correction frequencies dropped down to 10% in vivo [51] indicating that HDR is less active in long-term repopulating quiescent HSCs, which constitute a minor fraction of the HSPC population. Notably, all these studies reported the occurrence of incomplete HDR-driven repair. This resulted in the production of a relevant fraction of NHEJ-mediated InDels in the *HBB* gene, which can lead to β^S^-globin gene inactivation and the undesired generation of a β-thalassemic phenotype [52]. Interestingly, strategies to increase HDR efficiency in HSCs showed encouraging results and hold promise for a clinical application of gene correction strategies based on HDR [53,54].

HDR-based gene correction strategies have also been tested for correcting some β-thalassemia mutations. So far, most of the reported studies have been performed in patient-derived iPSCs and were focused on correcting specific disease-causing point mutations [55,56,57,58,59,60]. Interestingly, Cai and colleagues developed a strategy based on the targeted integration of a donor template containing the complementary DNA (cDNA) coding the wild-type *HBB* gene; this approach could potentially be applied to correct many β-thalassemia-causing mutations [61]. The major limitation of these strategies is the lack of a robust protocol for the generation of long-term engraftable HSCs from iPSCs.

### 4.2. NHEJ-Based Gene Correction Strategies

Although recent years have witnessed significant advances, the precise correction of causative mutations based on HDR remains an inefficient process in long-term repopulating HSCs. This is likely due to the poor activity of this DNA repair mechanism in quiescent cells. Thus, when applicable, imprecise disruption of DNA sequences by NHEJ is inherently more suitable for developing HSC-based therapeutic approaches. This is the case of specific genetic mutations affecting non-coding sequences of the *HBB* gene, such as the IVS1-110G.A and IVS2-654C.T β-thalassemia mutations, which generate de novo splice sites in *HBB* introns that leads to the formation of aberrant mRNAs and premature stop codons. In 2019, two independent groups reported an efficient TALEN- or CRISPR/Cas9-based editing strategy for disrupting the aberrant splice site generated by the IVS1-110G.A mutation in patient HSPCs [62,63] (Figure 1). This led to restoration of normal *HBB* splicing and increase in HbA expression in HSPC-derived RBCs. A similar strategy was used to target the IVS2-654C.T mutation [62].

## 5. Nuclease-Mediated Strategies for γ-Globin Reactivation

Elevated levels of HbF are associated with mild disease symptoms in both β-thalassemia and SCD patients [64]. In fact, HbF compensate for the HbA deficiency in β-thalassemia, whereas γ-globin exerts a potent antisickling effect in SCD. Therefore, reactivating HbF expression represents a universal strategy that can be effective for both SCD and β-thalassemia patients, allowing the development of single therapeutic product for treating both diseases, independently of the specific mutation.

Hereditary persistence of fetal hemoglobin (HPFH) is a benign genetic condition associated with high HbF levels in adult life. HPFH mutations are either large deletions or point mutations within the β-globin locus.

Large HPFH deletions are thought to eliminate HbF inhibitory sequences or juxtapose the γ-globin promoters to remote enhancer regions (Figure 1). First attempts using CRISPR/Cas9 to reproduce the naturally occurring Sicilian HPFH deletion have been performed by Ye et al. colleagues [65]. This 13-kb deletion in homozygosity resulted in a 2-fold increase in γ-globin mRNA expression in HSPC-derived erythroid clonal populations compared to untreated cells. A more detailed work studied HbF de-repression associated with large HPFH deletions by independently examining the contribution of an HPFH-like 13.6-kb deletion and smaller deletions encompassing a putative 3.5-kb γ-δ intergenic HbF silencer [66]. This work showed a robust HbF reactivation upon deletion or inversion of the 13.6-kb region in SCD HSPC-derived erythroid cells, while a mild γ-globin de-repression was obtained by disrupting only the putative HbF silencer, suggesting that alteration of the *HBB* locus architecture play a major role in deletional HPFH. However, the generation of large deletions requires the simultaneous use of two gRNAs, which might decrease the overall efficiency of genome editing and increase the occurrence of off-target editing and chromosomal rearrangements.

Genome editing strategies have also been developed to mimic HPFH mutations in the *HBG* promoters (Figure 1). Several HPFH mutations in the γ-globin promoters affect the binding of B cell CLL/lymphoma 11A (BCL11A) and leukemia/lymphoma related factor (LRF; also known as ZBTB7A or FBI-1), the two main γ-globin transcriptional repressors, thus leading to elevated γ-globin expression. Several nuclease-based editing approaches aimed at disrupting BCL11A and LRF binding sites (BSs) via the generation of Indels. The first study reproduced a naturally occurring 13-bp HPFH deletion (Δ13 bp) between −102 and −114 bp upstream of the transcription start site (TSS) of *HBG* genes [67]. Martyn and colleagues showed that Δ13 bp as well as HPFH point mutations mapping to the same region, impair the binding of BCL11A [68]. Disruption of the BCL11A BS induced a strong HbF reactivation with γ-globin transcripts accounting for up to 80% of the total β-like globin mRNAs in the human umbilical cord blood-derived erythroid progenitor (HUDEP-2) cell line [67]. A similar efficacy was proven in SCD HSPC-derived RBCs with the production of ~80% of F-cells and up to 35% of HbF over the total Hb types [69]. Interestingly, this work reported that approximatively half of the mutations were Δ13 bp deletions. This was likely due to the presence of 8-nucleotide tandem repeats flanking the Cas9 cleavage site that induced MMEJ. Investigating the capability to edit quiescent HSCs through MMEJ is crucial to determine the clinical relevance of this strategy. Interestingly, BCL11A BS-edited cells were able to engraft and differentiate in immunodeficient mice [69] and the limited reduction in the frequency of Δ13 bp, did not affect γ-globin expression in ex vivo generated RBCs. Furthermore, a recent work based on autologous transplantation of CRISPR/Cas9-treated non-human primate (NHP) HSPCs reported the long-term engraftment of BCL11A BS-edited cells (18% of editing efficiency with >1-year follow up) with, however, a significant reduction of total editing frequency [70]. This led to a limited γ-globin reactivation (1–5% over the total β-like globin chains) that might not be sufficient to achieve therapeutically meaningful HbF levels. However, results of stable engraftment and detectable HbF are encouraging. Targeting of the BCL11A BS in the *HBG* promoters has also been achieved by in vivo transduction of murine mobilized HSCs by using a CD46-targeting nonintegrating adenoviral vector expressing CRISPR/Cas9 [71]. This vector can transduce cells expressing CD46, such as HSCs. This in vivo approach (based on the mobilization of HSCs in the peripheral blood, followed by the injection of the adenoviral vector) can potentially bypass the limitations associated to ex vivo gene therapy, such as the loss of stemness due to extensive cell manipulation (which limits cell engraftment), the requirement of a transplantation unit, and myeloablation. In particular, in this work, Li et al. proved the efficacy of this strategy by modifying up to 26.6% of HSCs in a transgenic mouse model harboring the entire human β-locus (β-YAC mice [72]). This frequency allowed γ-globin mRNA reactivation (~10-fold increase compared to the controls) and the production of γ-globin chains accounting for up to 13% of the total β-like globins [71]. Although promising, this approach has still some limitations: (1) it requires the enrichment of transduced HSCs in vivo by introducing a resistance gene in the backbone of the adenoviral vector, thus potentially limiting HSC diversity; (2) CD46 is present in many non-hematopoietic tissues and the selectivity for hematopoietic cells needs to be demonstrated in humans; and (3) adenoviral vectors can elicit a strong inflammatory response. However, future investigations will hopefully address these issues in order to extend the use of gene therapy approaches in geographic areas lacking BM transplantation centers.

An alternative approach proposed by our group aims at targeting the LRF BS located in the −200 region of the *HBG* promoters. This strategy resulted in a potent γ-globin induction and correction of SCD cell phenotype in vitro coupled with the persistence of good editing efficiency in vivo in HSCs engrafted in immunodeficient mice [73].

Many studies validated BCL11A as an important γ-globin transcriptional repressor [74,75,76]. In humans, *BCL11A* haploinsufficiency is associated with elevated HbF expression [77,78]. However, BCL11A is essential for HSC function and B-cell development [79,80]. Indeed, transplantation of *BCL11A* knockout HSPCs in a NHP model resulted in low persistence of genetically modified cells [81], showing detrimental effects associated with *BCL11A* inactivation in HSCs. Therefore, many efforts have been devoted to knock-down *BCL11A* only in the erythroid lineage as a therapeutic strategy for β-hemoglobinopathies. Interestingly, erythroid-specific inactivation of *BCL11A* ensures high-level, pancellular HbF expression, preventing disease manifestations in a β-YAC humanized mouse model of SCD [82]. Concordantly, LV-mediated *BCL11A* downregulation using a miR-embedded shRNA showed promising results in a phase 1/2 clinical trial [83]. Erythroid-specific enhancers in *BCL11A* intron 2 have been identified as new targets for the treatment of β-hemoglobinopathies [84]. Canver et al. showed that CRISPR/Cas9 inactivation of a GATA1 activator BS in one of the erythroid-specific *BCL11A* intronic enhancers, induced substantial HbF expression in RBCs without affecting erythropoiesis or *BCL11A* expression in other lineages [85] (Figure 1). Similar results were obtained using a ZFN-based approach [86]. Later studies reported that GATA1 BS-edited cells can efficiently engraft and undergo multilineage differentiation in immunodeficient mice [87,88,89]. Safety and efficacy of this approach has also been demonstrated in NHP transplantation experiments, although a decrease in the proportion of edited alleles was observed at the late time points (with frequencies of up to 29%) [90]. This effective strategy is in an advanced state of clinical development compared to the other genome editing approaches. Two phase 1/2 clinical trials based on CRISPR/Cas9-mediated *BCL11A* enhancer editing are currently ongoing (NCT03655678 and NCT03745287 for transfusion dependent β-thalassemia and SCD, respectively). Early clinical results in one β-thalassemia and one SCD patient have been recently reported [91]. The two patients showed a high editing efficiency (60% and 80% in nucleated peripheral blood cells and BM, respectively) that remained stable 18 and 12 months after transplantation, respectively. This led to pancellular HbF expression, transfusion-independence, and elimination of vaso-occlusive episodes in the patient affected by SCD. Updated data on these trials have recently been reported at the Annual American Society of Hematology meeting [92]. Seven β-thalassemia patients showed HbF levels ranging from 4 to 13.1 g/dL, while in 3 SCD patients HbF accounted for 31 to 48% of the total hemoglobin.

Recent works showed that the heme-regulated inhibitor HRI (also known as EIF2AK1) is involved in γ-globin repression [93,94]. The *HRI* gene encodes an erythroid-specific protein kinase responsible for the phosphorylation of eIF2α, which, in turn, modulates mRNA translation. In particular, HRI augments the levels of the transcription factor ATF4, which promotes *BCL11A* transcription and therefore HbF silencing. CRISPR/Cas9-mediated inactivation of *HRI* resulted in increased γ-globin expression in HUDEP-2 cells and primary HSPC-derived erythroid cells without altering their differentiation [93,95]. The HRI kinase, given its nature, will likely serve as a potent pharmacological target to raise HbF levels.

Other potential targets for pharmacological or genome editing-mediated therapeutic HbF induction could be the components of the nucleosome remodeling and deacetylase (NuRD) repressor complex that is involved in Hb silencing [96,97,98,99].

## 6. Base and Prime Editing Approaches for β-Hemoglobinopathies

The DSB-free base and prime editing systems hold promise to safely modify the genome for developing therapeutic approaches for β-hemoglobinopathies [100].

Compared to HDR-based strategies, base editing is a promising therapeutic tool to precisely correct genetic mutations as it can occur in quiescent cells, such as HSCs [101]. CBEs have been used to correct the β-thalassemia-causing *HBB* −28 (A > G) mutation in patients’ fibroblasts [102], erythroid precursors [103] and HSPCs [104]. In this last work, CBE and gRNA electroporation led to 68% of corrective C > T mutations in HSPC-derived erythroid cells, thus restoring β-globin expression.

Base editing-mediated A:T to T:A conversion is not possible; thus, the SCD-causing mutation (GAG → GTG) cannot be reverted by the current BEs. However, the GTG codon can be converted to a GCG triplet coding for an alanine. This mutation is present in the Makassar allele (HbG) and is non-pathogenic in both heterozygous and homozygous individuals [105,106] (Figure 1). Recently, Miller et al. showed that an ABE variant recognizing a NRCH-PAM can be used to induce this mutation in HEK293T cells harboring the SCD mutation, demonstrating the feasibility of base editing-mediated approaches targeting the SCD-causing mutation [107].

Base editing strategies have also been explored to reactivate HbF expression, either by recapitulating HPFH mutations in the *HBG1/2* promoters [108,109,110] or by targeting the GATA1 BS in the erythroid-specific *BCL11A* enhancer [111]. Recent works proved the efficacy of these strategies in HSPCs. In particular, ABE variants have been used in healthy donor HSPCs for generating the −198 T > C HPFH mutation in the *HBG1/2* promoters; this mutation creates a binding site for the transcriptional activator KLF1 (Figure 1). The frequency of base conversion reached up to 60% and led to a 3.5-fold increase in γ-globin expression in HSPC-derived erythrocytes [112]. Similarly, Wang et al. used an ABE to introduce the −115C > T and −114 C > T HPFH mutations disrupting the BCL11A BS in the *HBG* promoters. A potent γ-globin reactivation was observed following erythroid differentiation of edited HSPCs obtained from healthy donors and β-thalassemia patients [113]. Finally, CBE delivery into SCD and β-thalassemic HSPCs resulted in efficient (86.3–93.3%) editing of the GATA1 BS in the *BCL11A* enhancer (Figure 1). The HSPC-derived erythroid progeny exhibited high HbF levels (up to 32%), and amelioration of the pathological cell phenotype (reduced RBC sickling in SCD samples and improved α-/β-globin chain balance in β-thalassemic cells). Importantly, xenotransplantation experiments in immunodeficient mice showed that C > T editing can occur in bona fide human HSCs [104].

Interestingly, given its intrinsic DSB-free nature, multiplex base editing is possible, as it avoids the generation of large genomic rearrangements. Zeng et al. showed that a base editing approach simultaneously correcting the β-thalassemia-causing *HBB* −28 (A > G) mutation and disrupting the GATA1 BS in the *BCL11A* enhancer is feasible and allows a better correction of the β-thalassemic phenotype compared to individual strategies [104].

Finally, proof-of-principle for the treatment of SCD by PE was provided by correcting the disease-causing A > T transversion mutation in HEK293T cells harboring the SCD allele [41] (Figure 1).

## 7. Nuclease-Free Strategies for β-Hemoglobinopathies Based on Peptide Nucleic Acids

**Figure 2:**
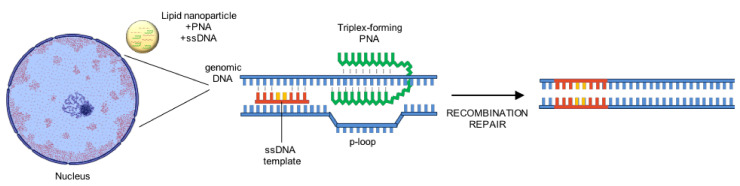
DSB-free insertion of genomic edits using PNAs. Lipid nanoparticle have been used to deliver PNA and single strand DNA (ssDNA) donor templates. After strand invasion by the PNA, the p-loop is recognized by the endogenous DNA repair machinery, which induce the introduction of the desired edits carried by the ssDNA. DSB: double strand break; PNA: peptide nucleic acid; ssDNA: single-stranded DNA.

Alternative nuclease-free strategies based on peptide nucleic acids (PNAs) have been developed to treat β-hemoglobinopathies. PNAs contain nucleobases with a peptide-like backbone, making them resistant to both proteases and nucleases, and conferring PNA/DNA complexes increased stability compared to DNA/DNA complexes due to the lack of negatively charged phosphodiester bonds. PNAs can form a triplex structure with a single strand of DNA, thus displacing the opposite strand that forms the so-called p-loop (Figure 2). This structure recruits the endogenous DNA repair machine, stimulating the insertion of short donor DNA fragments near to the PNA binding site via HDR. Importantly, this process does not generate Indels. However, PNAs do not readily cross the cellular membrane and are rapidly cleared within 10–30 min after intravenous or intraperitoneal administration; thus, a delivery vehicle is needed to achieve in vivo gene editing. Therefore, the PNAs and the donor DNA were delivered using nanoparticles in HSCs (Figure 2) using both ex vivo and in vivo gene therapy approaches to treat β-thalassemic mice carrying the human β-globin gene containing the β-thalassemia mutation IVS2-654 [114]. In particular, in vivo treatment of β-thalassemic mice resulted in up to 7% of gene correction in HSCs, with elevation of Hb levels and correction of the β-thalassemia phenotype. In utero delivery of nanoparticles containing PNAs and DNA donors was able to correct the IVS2-654 β-thalassemia mutation in the fetus, ensuring persistent postnatal amelioration of the disease with ~10% of edited alleles in adult BM HSPCs [115]. PNA/donor DNA treatment also yielded genome editing in human HSCs at a frequency of 3.4% [114]. Interestingly, off-target activity was minimal in both murine and human cells. Further studies are required to increase the editing efficiency in human HSCs (which is known to be refractory to HDR-mediated gene correction) and achieve a therapeutic benefit.

## 8. Off-Target Activity of Genome Editing Systems

Genome editing strategies hold promise to specifically modify a target genomic locus. However, the potential off-target DNA activity needs to be considered especially when developing therapeutic strategies. Methods for detecting off-targets have been recently reviewed [116,117]. Briefly, off-targets of designer nucleases can be predicted using in silico methods, or identified using in vitro or cell-based assays, which can detect DSBs in a genome-wide manner. In cellular assays are generally believed to be more reliable [118]; however, in most of the cases, surrogate cell lines need to be used because these methods have not been adapted yet to clinically relevant cells, such as HSPCs. As base and prime editors are characterized by little or no DSB generation, the assays off-targets to detect are more limited compared to designer nucleases, but they will probably be developed in the next future [116,117]. Finally, RNAseq is commonly used to detect RNA off-target activity of base editors [100].

Studies on genome editing approaches for β-hemoglobinopathies have used a variety of methods to evaluate the potential off-target activity [51,57,59,70,73]. Importantly, several works showed that the use of high-fidelity enzymes can substantially reduce off-target activity, while maintaining a good on-target editing efficiency [51,100].

## 9. Conclusions

Genome editing technologies provided a fertile soil for the development of novel therapeutic strategies for β-hemoglobinopathies. The last years have witnessed the development of an increasing number of technologies, experimental models and genetic targets for genome editing-based therapies. The study of cellular mechanisms allowing gene correction and regulatory mechanisms underlying Hb switching opened the way for the preclinical and clinical application of novel gene therapy approaches, which could become in the future a widespread therapeutic option for the high number of patients affected by β-hemoglobinopathies.

However, while many therapeutic targets have been identified and, for some strategies, early clinical data are very encouraging [83,91,92], the genome editing technologies still face some challenges. A first risk is the off-target effects on DNA and on both DNA and RNA in the case of BEs. The transient expression of genome editing tools and the use of more precise enzymes can substantially reduce off-target effects [103,112]. However, even minimal off-target activity needs to be closely monitored in both pre-clinical and clinical studies, as gene therapy approaches are based on the injection of a large number of HSPCs.

Similarly, HSPC viability, proliferation, engraftment and genome integrity must be carefully evaluated especially when using nuclease-based strategies that can affect cellular fitness and functionality [36], and generate large genomic rearrangements [34,35,37]. However, amelioration of culture conditions and delivery methods (e.g., usage of ssODN instead of AAV6 as donor template), and the use of highly specific editing tools can substantially reduce cytotoxicity [39] and the formation of genomic rearrangements, such as translocations between on- and off-target regions.

The novel BE and PE tools virtually abolish the risk of DSB-induced cytotoxicity and large rearrangements. However, while HSPC electroporation with nucleases has been established in many labs and is already employed in clinical trials, delivery of the large BE and PE enzymes is still challenging in primary cells.

Finally, pre-existing immunity against Cas9 or *de novo* immune responses to Cas9 should be taken in consideration; however, the transient expression of the CRISPR/Cas9-based tools likely minimizes the risk of elimination of genetically modified HSPCs by the immune system.

Despite these challenges, genome editing approaches hold promise for curing patients affected β-hemoglobinopathies. Nowadays, LV-mediated gene therapy strategies for β-hemoglobinopathies represent an established curative option. However, it is important to note that LVs are extremely expensive to manufacture (>300,000 euros per patient [119]). Most of the genome-editing approaches require the delivery of RNA/protein reagents that are likely less expensive thus, it is reasonable to suppose that they will replace LV-based gene therapy strategies in the coming years.

## Data Availability

Not applicable.
